# Measurement properties of brief neuropathy screening items in cancer patients receiving taxanes, platinums, or proteasome inhibitors

**DOI:** 10.1186/s41687-021-00377-z

**Published:** 2021-09-26

**Authors:** Robert Knoerl, Emanuele Mazzola, Sandra A. Mitchell, Fangxin Hong, Elahe Salehi, Nadine McCleary, Jennifer A. Ligibel, Kaitlen Reyes, Donna L. Berry

**Affiliations:** 1 Phyllis F. Cantor Center for Research in Nursing and Patient Care Services, Dana-Farber Cancer Institute, Boston, MA USA; 2 Present Address: University of Michigan School of Nursing, Ann Arbor, MI USA; 3 Department of Data Sciences, Dana-Farber Cancer Institute, Boston, MA USA; 4 Outcomes Research Branch, Division of Cancer Control and Population Sciences, National Cancer Institute, Rockville, MD USA; 5 Medical Oncology, Dana-Farber Cancer Institute, Boston, MA USA; 6 Biobehavioral Nursing and Health Informatics, University of Washington, Seattle, WA USA

**Keywords:** Patient-reported outcome measures, Chemotherapy-induced peripheral neuropathy, Psychometrics, Reliability, Validity, Patient-Reported Outcomes Version of the Common Terminology Criteria for Adverse Events

## Abstract

**Background:**

Timely detection of chemotherapy-induced peripheral neuropathy (CIPN) is critical to effectively tailor chemotherapy dose levels and offer supportive care. The purpose of this secondary analysis was to determine the reliability and validity of the two Patient-Reported Outcomes Version of the Common Terminology Criteria for Adverse Events (PRO-CTCAE™) numbness and tingling severity and interference items to screen for CIPN in patients receiving taxanes, platinums, or proteasome inhibitors.

**Methods:**

Participants (*N* = 142) completed the two PRO-CTCAE items, a 0–10 numerical rating scale of worst CIPN pain intensity, and the Quality of Life Questionnaire–CIPN20 (QLQ-CIPN20) prior to three clinical visits (T1, T2, T3) during neurotoxic chemotherapy. Participants completed the two PRO-CTCAE items again following the T3 clinical visit (T4). In addition, study staff administered the modified Total Neuropathy Score–Clinical Version (TNSc©) at T3. We examined floor (i.e., no CIPN severity or interference) and ceiling effects, test–retest reliability, concurrent validity, longitudinal validity, construct validity of the response categories, and sensitivity and specificity of the two PRO-CTCAE items.

**Results:**

At T3, 29% of participants had PRO-CTCAE severity scores at the floor; 60.1% of participants reported interference item scores at the floor. Agreements between scores reported at T3 and T4 for PRO-CTCAE severity (*ICC* = 0.79) and interference (*ICC* = 0.73) were moderate to strong. The PRO-CTCAE severity and interference items correlated moderately-strongly with QLQ-CIPN20 sensory (Spearman’s *ρ-range* = 0.53–0.72) and motor (Spearman’s *ρ-range* = 0.50–0.58) subscale scores. The Cohen’s *d* from T1 to T3 for the PRO-CTCAE items were small (severity: *d* = 0.32, interference: *d* = 0.40) and comparable to the effect sizes for change observed with the QLQ-CIPN20. The PRO-CTCAE severity (0–3) and interference (0–2) response categories distinguished respondents with significantly different levels of QLQ-CIPN20 sensory and motor subscale scores (*p* < 0.001 via Jonckheere-Terpstra tests). The sensitivity and specificity of the PRO-CTCAE severity item (cutoff > 0) to detect probable sensory peripheral neuropathy were 95.83% and 65.22%, while the sensitivity and specificity of the PRO-CTCAE™ interference item (cutoff > 0) were 51.39% and 73.91%.

**Conclusion:**

Preliminary evidence supports the reliability and validity of the PRO-CTCAE numbness and tingling items for CIPN screening, although there may be floor effects and limitations in the capacity of the PRO-CTCAE items to identify the full range of CIPN sensory and motor features beyond numbness and tingling.

*Trial Registration* ClinicalTrials.Gov, NCT03514680. Registered 21 April 2018. https://clinicaltrials.gov/ct2/show/NCT03514680

## Introduction

Chemotherapy induced peripheral neuropathy (CIPN) is a dose-dependent side effect of taxanes, platinums, and proteasome inhibitors that may necessitate the reduction or withdrawal of chemotherapy [1, 2] due to sensory (e.g., numbness, tingling, or pain in a stocking-glove pattern) or motor symptoms (e.g., weakness or cramps in the extremities) [3]. Sensory or motor CIPN symptoms may interfere with activities of daily living (e.g., walking, driving, typing on a computer) [4, 5] and increase risk of falls [6]. Post-treatment, CIPN symptoms may linger for months to years after and in some cases become permanent. Currently, there is only one recommended treatment for the symptomatic management of established CIPN symptoms (i.e., duloxetine) and no recommended prevention modalities [7].

Due to the lack of recommended treatments, precise and timely detection of CIPN is critical during neurotoxic cancer therapy treatment to effectively tailor therapy dosages. CIPN is most commonly measured in practice and research [8] using clinician-rated grading scales such as the National Cancer Institute Common Terminology Criteria for Adverse Events (CTCAE, now in version 5.0) [9]. However, clinician grading of CIPN using the CTCAE has demonstrated floor effects [10, 11] and low concurrent validity with CIPN experiences captured using patient-reported outcome measures [10]. Further, while there are a plethora of CIPN measures available, burdensome administration procedures (e.g., long survey measures; skill and time required by clinicians to complete objective tests) often complicate their use in practice [8, 12–14]. The difficulties of consistent implementation of CIPN evaluation in practice is highlighted by evidence suggesting that CIPN assessments are documented by clinicians in only 46% to 58.3% of their clinical encounters with patients receiving neurotoxic chemotherapy [12, 15].

To address the limitations that may be associated with clinician-rated or lengthy self-report measures of CIPN, brief (≤ 3 items) CIPN instruments with strong measurement properties are needed to improve the identification of CIPN in clinical practice [8]. The Patient-Reported Outcomes version of the Common Terminology Criteria for Adverse Events (PRO-CTCAE™) [16] numbness and tingling severity item and PRO-CTCAE numbness and tingling interference item are promising CIPN screening tools. The psychometric properties of the PRO-CTCAE library (78 symptoms) has been extensively evaluated in patients with cancer undergoing chemotherapy and/or radiation [17–19]. In addition, several studies support the concurrent validity of the PRO-CTCAE numbness and tingling items with PRO measures that include the European Organisation for Research and Treatment of Cancer Quality of Life Questionnaire-CIPN20 (QLQ-CIPN20) [11, 20] (e.g., *r* ≥ 0.55 for all comparisons between PRO-CTCAE numbness and tingling items and the QLQ-CIPN20 sensory and motor subscales) [11]; the Functional Assessment of Cancer Therapy/Gynecologic Oncology Group-Neurotoxicity questionnaire (FACT/GOG-Ntx) (Spearman’s *ρ* = 0.75; severity item only) [21]; and with clinician-rated measures such as the Total Neuropathy Score©-Reduced (Spearman’s *ρ* = 0.56; severity item only) [21]. Moreover, the PRO-CTCAE numbness and tingling items have demonstrated the capacity to detect CIPN-related interference earlier in chemotherapy treatment than the CTCAE [20]. The PRO-CTCAE numbness and tingling severity item has also been shown to discriminate patients with mild/no neuropathy and moderate-severe (Grade 2/3) neuropathy [21].

While the PRO-CTCAE numbness and tingling items show promising measurement properties [17–19], to our knowledge, the test–retest reliability and longitudinal validity of the severity and interference items have had limited testing in individuals receiving neurotoxic cancer therapy. Further, psychometric testing of the interference item has been limited to concurrent validity with the QLQ-CIPN20 or CTCAE in patients receiving taxanes or platinums [11, 20]. The purpose of this analysis was to determine the reliability and validity of the PRO-CTCAE numbness and tingling items as screening measures of CIPN in practice. Specifically, we estimated the floor and ceiling effects, test–retest reliability, concurrent validity, longitudinal validity, construct validity of the response categories, and sensitivity and specificity of the PRO-CTCAE numbness and tingling severity and interference items in cancer patients receiving taxanes, platinums, or proteasome inhibitors.

## Materials and methods

### Design, sample, and setting

The data for this secondary analysis were derived from a two-phase, longitudinal trial designed to explore the impact of a clinician decision support algorithm on clinicians’ documentation of CIPN assessment and management [22]. The sample consisted of 142 English speaking patients with breast or gastrointestinal cancer, or multiple myeloma who had received ≥ one infusion of neurotoxic therapy (e.g., taxanes, platinums, or proteasome inhibitors) at the time of consent and were scheduled to receive ≥ three more cycles of neurotoxic therapy. Patients were excluded if they had pre-existing neuropathy unrelated to cancer therapy. All participants were recruited from a National Cancer Institute-Designated Cancer Center. Approximately half of the sample (*n* = 70) participated in the usual care phase and the other half of the sample participated in the algorithm phase (*n* = 72). Clinicians received a CIPN assessment and management algorithm prior to each clinical visit for all participants during the algorithm phase. The following describes the measures, procedures, and statistical analyses pertinent to this secondary analysis of the measurement properties of the two PRO-CTCAE numbness and tingling severity and interference items.

### Measures

*PRO-CTCAE Numbness and Tingling Severity and Interference Items* The PRO-CTCAE Measurement System is comprised of an item library with 124 PRO items that evaluate the presence, frequency, severity, or interference of 78 cancer treatment-related symptomatic adverse events [16]. The two PRO-CTCAE items that pertain to CIPN evaluate the severity at its worst and the associated interference of numbness and tingling in the hands and feet over the past seven days. PRO-CTCAE numbness and tingling item responses are scored from 0 to 4 with higher scores reflecting greater severity and interference, respectively [17–19].

*5-Item Total Neuropathy Score – Clinical (TNSc©)* The 5-item TNSc© [23–25] is a measure that includes patient self-report of sensory (i.e., numbness, tingling, and pain [burning, aching, stabbing]) and motor (e.g., difficulty buttoning or climbing steps) neuropathy symptom severity and/or location questions. Both of these questions overlap with the numbness and tingling severity and interference items of the PRO-CTCAE, respectively. These self-reports are integrated with examiner-administered tests of vibration sensibility, strength, and deep tendon reflexes. Items are scored from 0 to 4 with total scores ranging from 0 to 20 (higher scores reflect more severe neuropathy). Several studies support the reliability and validity of the TNSc© to capture peripheral neuropathy in adults undergoing neurotoxic cancer treatment [26–28].

*0–10 Numerical Rating Scale (NRS) of Worst CIPN Pain Intensity* Worst CIPN pain intensity over the past seven days was quantified using a 0–10 NRS [29, 30]. Higher scores on the NRS represent more severe CIPN pain intensity.

*European Organization of Research and Treatment of Cancer QLQ-CIPN20 Sensory and Motor Subscales* The QLQ-CIPN20 sensory subscale consists of nine self-report items that measure on a four-point scale the severity of neuropathic symptoms in the hands or feet over the past seven days, while the motor subscale consists of eight-items that measure on a four-point scale CIPN-related functional deficits over the past seven days. Each subscale is summed and linearly transformed to a score that can range from 0 to 100, where higher scores represent greater CIPN symptom severity [31]. The QLQ-CIPN20 sensory subscale contains four questions (i.e., numbness or tingling in the hands and/or feet, respectively) that are similar in nature to the one-item PRO-CTCAE numbness and tingling severity item. The QLQ-CIPN20 motor subscale contains five questions that are similar in nature (e.g., ask about specific functional limitations associated with CIPN such as walking, holding a pen, opening jars) to the one-item PRO-CTCAE numbness and tingling interference item. Evidence supports the internal consistency reliability (e.g., Cronbach’s alpha: 0.88 for sensory and motor subscales, respectively), concurrent validity, and responsiveness to change of the QLQ-CIPN20 sensory and motor subscales [10, 32]. In this study, the three-item autonomic symptom subscale was not administered due to its low item-item correlations with the rest of the QLQ-CIPN20 [10].

### Procedures

Participants completed the PRO-CTCAE numbness and tingling items, 0–10 NRS of worst CIPN pain intensity, and QLQ-CIPN20 using an iPad at the cancer center before each of three consecutive clinical visits (i.e., T1, T2, T3) during neurotoxic therapy or up to approximately one month after neurotoxic cancer therapy completion. In addition, a trained member of the study staff administered the TNSc© at T3. Four study staff members, including the principal investigator (RK) administered the TNSc©. On the same day as the T3 visit, the PRO-CTCAE numbness and tingling items were administered again (T4) to evaluate test–retest reliability. The T4 surveys were administered after the provider visit while the participants were in the waiting room prior to receiving chemotherapy or when the participants were actively receiving chemotherapy. At the conclusion of study-related procedures, study staff abstracted cancer treatment-related information from participants’ medical records.

### Statistical analyses

Data from participants in both study phases were pooled for this psychometric analysis. Due to the dose-dependent nature of CIPN, unless otherwise specified, all analyses were evaluated using data from T3, the time point when participants were anticipated to have received the greatest cumulative dosage of neurotoxic therapy. Descriptive statistics were calculated, and distributions were inspected for proportions at floor and ceiling. Floor and ceiling effects were calculated by frequencies and proportions of respondents reporting the lowest or highest possible scores.

Test–retest reliability of PRO-CTCAE numbness and tingling items between T3 and T4 was calculated in a subset of participants using the intraclass correlation coefficient (ICC) based on a two-way mixed effects analysis of variance model with interaction for the absolute agreement between single scores [33]. Values less than 0.50, between 0.50 and 0.75, between 0.75 and 0.90, and greater than 0.90 were interpreted as poor, moderate, good, and excellent reliability, respectively [34]. Longitudinal validity refers to how sensitive a measure is in detecting the real underlying change in symptom severity over time [35]. To evaluate longitudinal validity, a Cohen’s *d* effect size was calculated reflecting changes from T1 to T3 in PRO-CTAE numbness and tingling items, 0–10 NRS of worst CIPN pain intensity, and QLQ-CIPN20 sensory and motor subscale scores.

Concurrent validity between the PRO-CTCAE numbness and tingling items, and the QLQ-CIPN20 sensory and motor subscales, the 0–10 NRS of worst CIPN pain intensity, and the TNSc© was assessed using Spearman’s correlation.

Construct validity of the PRO-CTCAE numbness and tingling item response categories was evaluated with a one-sided Jonckheere-Terpstra Test [36] for ordered differences. The purpose of this analysis was to determine if there was monotonic ordering of scores on the QLQ-CIPN20 sensory and motor subscale, 0–10 NRS of worst CIPN pain intensity, or TNSc© across the item response categories for the PRO-CTCAE severity (0, 1, 2, and ≥ 3, respectively) and interference (0, 1, and ≥ 2, respectively) items. PRO-CTCAE severity item scores ≥ 3 and PRO-CTCAE interference item scores ≥ 2 were collapsed as few individuals had scores at or near the top of the scoring range (0–4).

Sensitivity refers to a screening measure’s ability to accurately identify a patient with the disease, while specificity refers to a screening measure’s ability to accurately identify a participant without the disease (higher values of both metrics are desirable) [37]. The sensitivity and specificity of the PRO-CTCAE numbness and tingling items were evaluated at cutoff levels of zero for each item in comparison to the reference definition of “probable sensory peripheral neuropathy” that is used for diabetic peripheral neuropathy [38]. The definition of probable sensory peripheral neuropathy requires participants to have two of the following signs or symptoms: neuropathic symptoms (e.g., numbness, tingling, or pain), decreased distal sensation, and/or decreased ankle reflexes [38]. Thus, participants with TNSc© Sensory scores ≥ 1 and either TNSc© Sensory Function: Vibration Sensibility scores ≥ 1 or TNSc© Reflexes scores ≥ 1 were classified as exhibiting probable sensory peripheral neuropathy. The PRO-CTCAE numbness and tingling items also were evaluated for their sensitivity and specificity to detect painful CIPN at cutoff levels of zero for each item. For the reference measure, 0–10 NRS of worst CIPN pain intensity scores ≥ 4/10 were interpreted as indicating the presence of painful CIPN, while scores < 4/10 indicated the absence of painful CIPN [39]. All analyses were conducted with R Statistical Software and evaluated at a significance level of 0.05.

## Results

### Sample characteristics

Data from 142 participants were pooled for these psychometric analyses (*n* = 70 usual care phase, *n* = 72 algorithm phase). In summary, participants were a median of 57 (*Range*: 27–80) years old, and predominantly female (66%), Caucasian (90%), received undergraduate (33.1%) or post graduate degree training (32.4%), working full-time (38%), diagnosed with a gastrointestinal (51%) or breast (37%) malignancy or multiple myeloma (11%), and receiving platinum (50%) (e.g., common treatment for gastrointestinal malignancies), taxane (35.9%) (e.g., common treatment for breast cancer), or proteasome-inhibitor-based (11%) cancer therapy (e.g., common treatment for multiple myeloma). Approximately 56% of the sample had received ≥ two-thirds of their planned course of neurotoxic therapy by T3 [22].

### Response distributions of the PRO-CTCAE numbness and tingling items

Table 1 presents the summary statistics for the QLQ-CIPN20 sensory and motor subscales, PRO-CTCAE™ numbness and tingling items, 0–10 NRS of worst CIPN pain intensity, and TNSc© (T3 only) from T1 to T3. Overall, mean scores for all CIPN measures increased over time reflecting worsening CIPN severity. At T3 (*n* = 138), approximately 29% of participants reported PRO-CTCAE™ severity item scores at the floor (score = 0) and approximately 60% of participants reported PRO-CTCAE™ interference item scores (score = 0) at the floor. Only one participant reported a PRO-CTCAE™ severity item score at the ceiling (severity = 4, interference = 2).

**Table 1:** QLQ-CIPN20, PRO-CTCAE™, and TNSc© Sample statistics at each study visit

Measure	*Mean* (*SD)*	*Median* (*range*)	Proportion at floor (%)	Proportion at ceiling (%)
*PRO-CTCAE numbness and tingling severity*				
T1 (*n* = 140)	0.79 (0.78)	1 (0–3)	57 (40.7%)	0
T2 (*n* = 137)	0.91 (0.84)	1 (0–3)	50 (36.5%)	0
T3 (*n* = 138)	1.08 (0.88)	1 (0–4)	40 (29%)	1 (0.07%)
T4 (*n* = 123)	1.03 (0.83)	1 (0–3)	35 (28.5%)	0
*PRO-CTCAE numbness and tingling interference*				
T1 (*n* = 139)	0.25 (0.54)	0 (0–2)	111 (79.9%)	0
T2 (*n* = 140)	0.49 (0.79)	0 (0–3)	93 (66.4%)	0
T3 (*n* = 138)	0.55 (0.76)	0 (0–3)	83 (60.1%)	0
T4 (*n* = 126)	0.52 (0.72)	0 (0–3)	76 (60.3%)	0
*QLQ-CIPN20 sensory*				
T1 (*n* = 140)	7.84 (10.51)	3.70 (0–55.56	56 (40%)	0
T2 (*n* = 140)	11.02 (11.02)	7.41 (0–55.56)	33 (23.6%)	0
T3 (*n* = 138)	11.92 (11.88)	7.41 (0–55.56)	33 (23.9%)	0
*QLQ-CIPN20 motor*				
T1 (*n* = 140)	5.15 (7.60)	0 (0–37.5)	75 (53.6%)	0
T2 (*n* = 139)	7.61 (9.74)	4.17 (0–50)	58 (41.7%)	0
T3 (*n* = 138)	8.49 (10.57)	4.17 (0–54.17)	54 (39.1%)	0
*0*–*10 NRS of worst CIPN pain Intensity*				
T1 (*n* = 138)	1.41 (1.98)	0 (0–9)	71 (51.4%)	0
T2 (*n* = 139)	1.99 (2.41)	1 (0–10)	61 (43.9%)	0
T3 (*n* = 138)	1.97 (2.16)	1 (0–9)	58 (42%)	0
*TNSc©*				
T3 (*n* = 118)	6.75 (2.9)	7 (1–13)	0	0

Table describes sample statistics for PRO-CTCAE item (*Range* = 0–4) QLQ-CIPN20 sensory and motor subscale (*Range* = 0–100), 0–10 NRS of worst CIPN pain intensity (*Range* = 0–10) and TNSc© (*Range* = 0–20) scores at each study time point (T1–T4). Higher scores on all measures represent worse CIPN severity

CIPN-Chemotherapy-Induced Peripheral Neuropathy, PRO-CTCAE™—Patient-Reported Outcomes Version of the Common Terminology Criteria for Adverse Events, QLQ-CIPN20-Quality of Life Questionnaire-Chemotherapy-Induced Peripheral Neuropathy Scale, TNSc©—Total Neuropathy Score-Clinical Version

### Test–retest reliability

The median time elapsed between the T3 and T4 administration of the PRO-CTCAE™ numbness and tingling items was 131 min (*Range* = 1–398). Agreement between T3 and T4 PRO-CTCAE™ numbness and tingling item scores was moderate to strong [40] (severity: *Adjusted ICC* = 0.79; interference: *Adjusted ICC* = 0.73, *n* = 123). Agreement between T3 and T4 PRO-CTCAE™ numbness and tingling item scores diminished when we restricted the sample to only individuals who scored a “1” or higher at T3 on either item (severity: *Adjusted ICC* = 0.52, *n* = 82; interference: *Adjusted ICC* = 0.50, *n* = 45).

### Concurrent validity

Table 2 presents the bivariate correlations among PRO-CTCAE numbness and tingling items, QLQ-CIPN20 sensory and motor subscale, 0–10 NRS of worst CIPN pain intensity, and TNSc© scores at T3. PRO-CTCAE numbness and tingling severity and interference item scores showed only moderate correlation with one another (Spearman’s *ρ* = 0.59), suggesting that these two items evaluate different aspects of the symptom experience. PRO-CTCAE severity item scores were highly correlated (Spearman’s *ρ* = 0.72) with QLQ-CIPN20 sensory subscale scores, and moderately correlated with QLQ-CIPN20 motor subscale (Spearman’s *ρ* = 0.50) and 0–10 worst CIPN pain intensity scores (Spearman’s *ρ* = 0.65). Correlations were low between the TNSc© and the PRO-CTCAE severity (Spearman’s *ρ* = 0.48) and interference (Spearman’s *ρ* = 0.30) items. PRO-CTCAE interference item scores were moderately correlated with QLQ-CIPN20 sensory (Spearman’s *ρ* = 0.53) and motor subscales (Spearman’s *ρ* = 0.58), and with the 0–10 worst CIPN pain intensity NRS scores (Spearman’s *ρ* = 0.55).

**Table 2:** Correlations among QLQ-CIPN20, PRO-CTCAE numbness and tingling items, TNSc©, and worst CIPN pain intensity scores

Measure	PRO-CTCAE numbness and tingling items		0–10 worst CIPN pain intensity	QLQ-CIPN20		TNSc©
	Severity	Interference		Sensory	Motor	
PRO-CTCAE Severity	1	0.59	0.65	0.72	0.50	0.48
PRO-CTCAE Interference		1	0.55	0.53	0.58	0.30
0–10 Worst CIPN Pain Intensity			1	0.56	0.40	0.27
QLQ-CIPN20 Sensory				1	0.56	0.48
QLQ-CIPN20 Motor					1	0.40
TNSc©						1

Table describes correlations among the PRO-CTCAE Numbness and Tingling Severity and Interference Items, QLQ-CIPN20, 0–10 worst CIPN pain intensity NRS (*n* = 138), and TNSc© (*n* = 118) at T3

CIPN-Chemotherapy-Induced Peripheral Neuropathy, PRO-CTCAE™—Patient-Reported Outcomes Version of the Common Terminology Criteria for Adverse Events, QLQ-CIPN20-Quality of Life Questionnaire-Chemotherapy-Induced Peripheral Neuropathy Scale, TNSc©—Total Neuropathy Score-Clinical Version

### Longitudinal validity

The average time elapsed between T1 and T3 was 41.43 days (*Range* = 14–149). The Cohen’s *d* for change in PRO-CTCAE numbness and tingling item scores (severity: *d* = 0.32, 95% *CI* = -0.02, 0.66); interference: *d* = 0.40, 95% *CI* = 0.06, 0.75) and QLQ-CIPN20 sensory (*d* = 0.41, 95% *CI* = 0.07, 0.76) and motor (*d* = 0.38, 95% *CI* = 0.04, 0.72) subscale scores from T1 to T3 were small. The Cohen’s *d* for change in 0–10 NRS of worst CIPN pain intensity scores was the smallest (*d* = 0.28, 95% *CI* = − 0.07, 0.62).

### Construct validity of PRO-CTCAE numbness and tingling items’ response categories

**Fig. 1:**
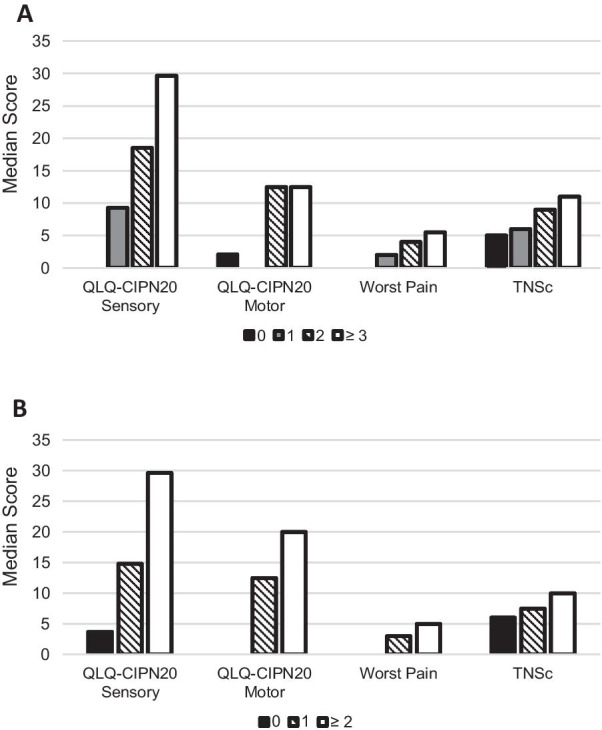
Median CIPN patient-reported and composite measure scores by PRO-CTCAE item response category at T3. **a** PRO-CTCAE numbness and tingling severity item. **a** Differences in median QLQ-CIPN20 Sensory, QLQ-CIPN20 Motor, 0–10 worst CIPN pain intensity NRS (*n* = 138), and TNSc© scores (*n* = 118) by PRO-CTCAE Numbness and Tingling Severity Item Score at T3. Severity = 0; TNSc© *n* = 33; QLQ-CIPN20 and worst pain measures *n* = 40. Severity = 1; TNSc© *n* = 49; QLQ-CIPN20 and worst pain measures *n* = 54. Severity = 2; TNSc© *n* = 33; QLQ-CIPN20 and worst pain measures *n* = 38. Severity ≥ 3; TNSc© *n* = 3; QLQ-CIPN20 and worst pain measures *n* = 6. **b** PRO-CTCAE Numbness and Tingling Interference Item. **b** Differences in median QLQ-CIPN20 Sensory, QLQ-CIPN20 Motor, 0–10 worst CIPN pain intensity NRS (*n* = 138), and TNSc© scores (*n* = 118) by PRO-CTCAE Numbness and Tingling Interference Item Score at T3. Interference = 0; TNSc© *n* = 69; QLQ-CIPN20 and worst pain measures *n* = 83. Interference = 1; TNSc© *n* = 34; QLQ-CIPN20 and worst pain measures *n* = 36. Interference ≥ 2; TNSc© *n* = 15; QLQ-CIPN20 and worst pain measures *n* = 19

Figure 1 displays median QLQ-CIPN20 sensory and motor subscale, 0–10 worst CIPN pain intensity, and TNSc© scores, by increasing PRO-CTCAE numbness and tingling item scores at T3. The response categories of the PRO-CTCAE severity (i.e., 0 to ≥ 3 categories) and interference (i.e., 0 to ≥ 2 categories) items demonstrated strong construct validity. Increasing score levels of PRO-CTCAE severity and interference discriminated respondents with statistically significantly different QLQ-CIPN20 sensory subscale scores (severity: *JT* = 5644.5, *p* < 0.0001, interference: *JT* = 4255.5, *p* < 0.0001), and QLQ-CIPN20 motor subscale scores (severity: *JT* = 4550, *p* < 0.0001, interference: *JT* = 4177, *p* < 0.0001), as well as greater CIPN pain intensity (Severity: *JT* = 5338, *p* < 0.0001; interference: *JT* = 4248, *p* < 0.0001), and higher TNSc© scores (severity: *JT* = 3396.5, *p* < 0.0001; interference: *JT* = 2544, *p* = 0.0008).

### Sensitivity and specificity

Table 3 presents the sensitivity and specificity of the PRO-CTCAE numbness and tingling items to detect probable sensory peripheral neuropathy and painful CIPN. In discriminating probable sensory peripheral neuropathy, the PRO-CTCAE severity item demonstrated a sensitivity of 95.83% and a specificity of 65.22%, while the interference item demonstrated a sensitivity of 51.39% and specificity of 73.91%. The sensitivity and specificity of the PRO-CTCAE severity item to detect painful CIPN were 100% and 38.46%, respectively, while the sensitivity and specificity of the interference item for painful neuropathy were 76.47% and 72.12%.

**Table 3:** Sensitivity and specificity of the PRO-CTCAE items to probable sensory peripheral neuropathy and painful chemotherapy-induced peripheral neuropathy (CIPN)

	Probable sensory peripheral neuropathy + (*n* = 72)	Probable sensory peripheral neuropathy − (*n* = 46)
**Test** + PRO-CTCAE Severity > 0/4	69	16
**Test** − PRO-CTCAE Severity = 0/4	3	30
	Sensitivity = 95.83%	Specificity = 65.22%
**Test** + PRO-CTCAE Interference > 0/4	37	12
**Test** − PRO-CTCAE Interference = 0/4	35	34
	Sensitivity = 51.39%	Specificity = 73.91%

	Painful CIPN + (*n* = 34)	Painful CIPN − (*n* = 104)
**Test** + PRO-CTCAE Severity > 0/4	34	64
**Test** − PRO-CTCAE Severity = 0/4	0	40
	Sensitivity = 100%	Specificity = 38.46%
**Test** + PRO-CTCAE Interference > 0/4	26	29
**Test** − PRO-CTCAE Interference = 0/4	8	75
	Sensitivity = 76.47%	Specificity = 72.12%

Table describes the sensitivity and specificity of the PRO-CTCAE Numbness and Tingling Severity and Interference Items to probable sensory peripheral neuropathy (*n* = 118) or painful CIPN (*n* = 138) at T3. Participants who reported Total Neuropathy Score-Clinical Version (TNSc©) Sensory scores ≥ 1/4 and either TNSc© Sensory Function: Vibration Sensibility scores ≥ 1/4 or TNSc© Reflexes scores ≥ 1/4 were classified as exhibiting probable sensory peripheral neuropathy [38]. Participants who reported TNSc© Sensory scores = 0/4 were classified as not exhibiting probable sensory peripheral neuropathy. Participants who reported 0–10 numerical rating scale of worst CIPN pain intensity scores ≥ 4/10 over the past week were interpreted as indicating the presence of painful CIPN [39]. Participants who reported < 4/10 worst CIPN pain intensity over the past week were classified as not experiencing painful CIPN. Higher scores on all measures represented worse symptom severity

## Discussion

The purpose of this study was to evaluate the measurement properties of the PRO-CTCAE numbness and tingling severity and interference items for use in screening for CIPN in patients receiving taxanes, platinum-based agents, or proteasome inhibitors. Our results demonstrate several psychometric strengths of the PRO-CTCAE items to screen for CIPN, but also suggest areas where precision, sensitivity and specificity might be improved. The PRO-CTCAE severity item, but not the interference item, identified 95.83% of individuals with probable sensory peripheral neuropathy, although both items demonstrated only modest specificity [37]. For the detection of probable sensory peripheral neuropathy, our observations about the high sensitivity and modest specificity of the PRO-CTCAE items are encouraging. The adverse consequences of a false negative (i.e., low sensitivity) of mild CIPN may be considerable as the earliest detection of the onset or worsening of CIPN directs clinical management. A false positive (i.e., low specificity) in the setting of mild CIPN is not concerning as only continued close monitoring is warranted for mild CIPN. Further research to evaluate the sensitivity and specificity of PRO-CTCAE numbness and tingling item cutoff scores associated with more severe neuropathy is needed because a false positive of more severe CIPN during active treatment may prompt clinicians to dose-reduce neurotoxic chemotherapy to prevent worsening symptoms.

Although the test–retest reliabilities of the PRO-CTCAE numbness and tingling items were moderate to strong, these results should be interpreted with caution and replication in future studies is recommended. In sensitivity analyses, the test–retest reliability of the items decreased when participants who did not report CIPN (i.e., scores of 0) were excluded, suggesting limited variability in the sample. In addition, the test–retest reliability estimates may also have been inflated given the short duration between T3 and T4 survey administration. The PRO-CTCAE and QLQ-CIPN20 effect size estimates for change across three clinical visits were small and comparable. While the Cohen’s *d* observed for the QLQ-CIPN20 sensory and motor subscales in this study were lower than previously reported in patients receiving neurotoxic chemotherapy (i.e., *d* = 0.82 QLQ-CIPN20 sensory, *d* = 0.48 QLQ-CIPN20 motor) [10], our observations support a conclusion that the two PRO-CTCAE items have similar sensitivity in detecting changes in CIPN severity and interference between visits, compared to the sensory and motor subscales of the QLQ-CIPN20 measure.

Study results highlight several potential considerations in the implementation of the PRO-CTCAE numbness and tingling items for CIPN screening and evaluation in patients on cancer therapy. While there were no observed ceiling effects for PRO-CTCAE severity or interference item scores, at the same time, we did observe floor effects in the PRO-CTCAE severity and interference items scores, with proportions at the floor exceeding the threshold of 15% or more of the sample [41]. A similar observation has recently been reported by Nyrop et al. [1] who found that approximately 27% and 55% of women with breast cancer receiving neurotoxic chemotherapy reported scores (maximum at any point during treatment) at the floor of the Patient-Reported Symptom monitoring system form severity and interference item score ranges (similar format to PRO-CTCAE scoring system) (*N* = 184) [1]. It is possible that these observed floor effects are sample dependent. Consistent with that possibility, we noted that the response distributions for PRO-CTCAE and QLQ-CIPN20 scores were comparable. As CIPN is a dose-dependent phenomena, one possibility for the low CIPN severity observed in our sample is that only about half of the sample received at least 2/3 of their planned neurotoxic chemotherapy at T3. Replication in a larger and more diverse sample of patients receiving cancer treatments associated with CIPN is needed to confirm whether our observations are durable. Additional research may be conducted to test the content validity of the PRO-CTCAE numbness and tingling items in a sample of patients with CIPN to determine if changes to the terminology of the measure may be required for patients to more readily report mild CIPN. While we await the results of additional research, the interpretation of studies that utilize PRO-CTCAE should keep in mind that the two CIPN items may be unable to differentiate among respondents at the low end of the severity spectrum. Continued research to develop and test new approaches to screening for CIPN, particularly at its earliest onset, also appear warranted.

The correlations reported by Knoerl et al. [11] between the PRO-CTCAE severity and interference item scores and QLQ-CIPN20 sensory (*r* = 0.76, *r* = 0.78) and motor (*r* = 0.55, *r* = 0.77) subscale scores in a sample of women receiving paclitaxel [11] were higher than those observed in this study. The less favorable metrics of concurrent validity observed in this analysis may indicate that the phrasing of the PRO-CTCAE numbness and tingling items may not fully capture variability in the range of sensory experiences and functional limitations experienced by individuals with oxaliplatin-induced peripheral neuropathy (which often presents with increased muscle weakness or sensitivity to cold objects) [42] or bortezomib-induced peripheral neuropathy (which typically presents with neuropathic pain) [43]. At the same time, there is evidence that the PRO-CTCAE response categories distinguish respondents with significantly different levels of CIPN symptom severity. Our observations extend the results of McCrary et al. [21] who demonstrated that the PRO-CTCAE severity item response categories (i.e., 0– ≥ 3) were able to discriminate worsening FACT/GOG-Ntx (*p* < 0.001) scores [21].

Opportunities to improve the precision of the PRO-CTCAE items represent an important future direction. The modest correlations observed between the PRO-CTCAE items and the measures of painful neuropathy and motor weakness also suggest that the capacity of the PRO-CTCAE to distinguish CIPN features beyond numbness and tingling, including pain or motor weakness, may be constrained. Clinicians should assess CIPN pain intensity and motor weakness separately because the PRO-CTCAE severity and interference items do not contain phrasing specific to neuropathic pain or motor weakness. Similarly, PRO-CTCAE numbness and tingling item scores should not be used to guide management decisions for patients with painful CIPN. Future consideration should be given to expanding the PRO-CTCAE numbness and tingling items to include questions that reflect neuropathic pain to improve the precision and discrimination of the measure, as this is a common feature of CIPN during and following neurotoxic cancer therapy [44].

Several caveats should be considered in interpreting our findings. Power analyses were not conducted for the exploratory analyses described in this research. As such, some of our analyses may have been underpowered, and these analyses should be replicated in future samples. Our single-institution sample was also predominantly white, non-Hispanic, female, and older in age, thereby decreasing the external generalizability of our findings. At the same time, the diversity of the neurotoxic agents administered in this study strengthens the generalizability of our observations. Observations in this sample were obtained from participants who were at varying points in their course of neurotoxic therapy, and only about 56% of the sample had completed a majority of the treatment cycles in their planned course of therapy. As such, effect size estimates with respect to changes in CIPN symptom severity over time should be interpreted cautiously. In addition, CIPN symptom severity remained generally low, requiring us to collapse score categories for many of our analyses. While four different study staff members administered the TNSc©, methods to assess inter-rater reliability among the four raters were not implemented. The test–retest reliability analyses are limited by the short time period between survey administration. Lastly, the sensitivity and specificity analyses are limited by the lack of a gold standard measure as the reference measure of CIPN and by the low prevalence of severe CIPN among the sample [37]. At the same time, the case definition of probable sensory peripheral neuropathy used in our examination of sensitivity and specificity was derived from case definitions used for the identification of diabetic neuropathy [38] and emphasized the detection of mild symptoms.

## Conclusion

The results of this analysis contribute to the evidence base about the measurement properties of the PRO-CTCAE numbness and tingling severity and interference items in patients receiving neurotoxic cancer therapy. Preliminary evidence supports the reliability and validity of the PRO-CTCAE numbness and tingling items as screening measures of CIPN, although there may be opportunities to improve the precision and discrimination of the items to capture the earliest onset of CIPN and to reflect the full range of CIPN features.

## Data Availability

The datasets used and/or analyzed during the current study are available from the corresponding author on reasonable request.

## Abbreviations

CIPN: Chemotherapy-induced peripheral neuropathy
CTCAE: Common terminology criteria for adverse events
FACT/GOG- Ntx: Functional Assessment of Cancer Therapy/Gynecological Cancer Group Neurotoxicity Questionnaire
NRS: Numerical Rating Scale
PRO: Patient reported outcomes
PRO-CTCAE™: Patient-Reported Outcomes Version of the Common Terminology Criteria for Adverse Events
QLQ-CIPN20: Quality of Life Questionnaire-Chemotherapy-Induced Peripheral Neuropathy Scale
TNSc©: Total neuropathy score-clinical version
